# Different rates of spontaneous mutation of chloroplastic and nuclear viroids as determined by high-fidelity ultra-deep sequencing

**DOI:** 10.1371/journal.ppat.1006547

**Published:** 2017-09-14

**Authors:** Amparo López-Carrasco, Cristina Ballesteros, Vicente Sentandreu, Sonia Delgado, Selma Gago-Zachert, Ricardo Flores, Rafael Sanjuán

**Affiliations:** 1 Instituto de Biología Molecular y Celular de Plantas, Consejo Superior de Investigaciones Científicas-Universitat Politècnica de València, València, Spain; 2 Institute for Integrative Systems Biology (I2SysBio), Consejo Superior de Investigaciones Científicas-Universitat de València, València, Spain; 3 Servicio de Genómica, Universitat de València, València, Spain; 4 Department of Molecular Signal Processing, Leibniz Institute for Plant Biochemistry, Halle (Saale), Germany; 5 Departamento de Genética, Universitat de València, València, Spain; The University of Sydney, AUSTRALIA

## Abstract

Mutation rates vary by orders of magnitude across biological systems, being higher for simpler genomes. The simplest known genomes correspond to viroids, subviral plant replicons constituted by circular non-coding RNAs of few hundred bases. Previous work has revealed an extremely high mutation rate for chrysanthemum chlorotic mottle viroid, a chloroplast-replicating viroid. However, whether this is a general feature of viroids remains unclear. Here, we have used high-fidelity ultra-deep sequencing to determine the mutation rate in a common host (eggplant) of two viroids, each representative of one family: the chloroplastic eggplant latent viroid (ELVd, *Avsunviroidae*) and the nuclear potato spindle tuber viroid (PSTVd, *Pospiviroidae*). This revealed higher mutation frequencies in ELVd than in PSTVd, as well as marked differences in the types of mutations produced. Rates of spontaneous mutation, quantified *in vivo* using the lethal mutation method, ranged from 1/1000 to 1/800 for ELVd and from 1/7000 to 1/3800 for PSTVd depending on sequencing run. These results suggest that extremely high mutability is a common feature of chloroplastic viroids, whereas the mutation rates of PSTVd and potentially other nuclear viroids appear significantly lower and closer to those of some RNA viruses.

## Author summary

Spontaneous mutations are the ultimate source of genetic variation and their characterization provides fundamental information about evolutionary processes. The highest mutation rate so far described corresponds to a hammerhead viroid infecting plant chloroplasts. Viroids are plant-exclusive parasites constituted by 250–400 nt-long, non-protein-coding RNAs, and are divided into two families with distinct mechanisms of replication and localization: chloroplastic (*Avsunviroidae*), and nuclear (*Pospiviroidae*). Here, we have used high-fidelity ultra-deep sequencing to compare side by side the mutation rates of one representative member of each viroid family in the same host. We found that the mutation rate of the nuclear viroid was several fold lower than that of the chloroplastic viroid.

## Introduction

Spontaneous mutations are pivotal to evolution as they constitute the ultimate source of genetic variation. The biochemical and genetic bases of replication fidelity have been extensively studied, and it is well-established that spontaneous mutation rates vary by orders of magnitude across biological systems [1, 2]. Whereas bacteria and other microorganisms show highly accurate replication, RNA viruses replicate with frequent errors [3]. Yet, the lowest replication fidelity reported so far corresponds to chrysanthemum chlorotic mottle viroid (CChMVd), a chloroplastic viroid in which a mutation is incorporated approximately every 400 bases copied [4]. Viroids are small (250–400 nt), circular, highly-structured RNAs that do not encode proteins and are copied by nuclear or chloroplastic DNA-dependent RNA polymerases forced to accept RNA templates [5–7]. They infect plants exclusively and their pathogenicity has been linked to RNA silencing [6], although other mechanisms cannot be excluded. Chloroplastic viroids encode in both polarity strands hammerhead ribozymes that play an essential role in their replication cycle. Together with their extreme simplicity, the presence of ribozymes makes these viroids reminiscent of the primordial replicons postulated by the RNA world hypothesis for the origin of life [5, 8].

**Fig 1:**
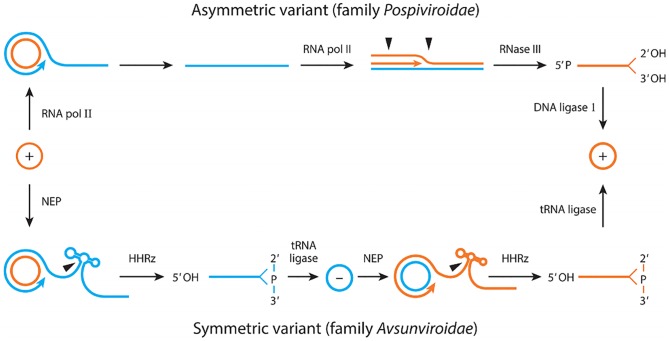
Mechanism proposed for replication of viroids. The asymmetric pathway with one rolling-circle (upper) is followed by members of the family *Pospiviroidae*, while the symmetric pathway with two rolling-circles (lower) is followed by members of the family *Avsunviroidae*. Orange and blue lines refer to plus (+) and minus (-) polarities, respectively and processing sites are marked by arrowheads. The enzymes and ribozymes that catalyze the replication steps are indicated. Notice that RNA polymerase II (and NEP) is redirected to transcribe RNA templates and DNA ligase I to circularize RNA substrates. Abbreviations: HHRz, hammerhead ribozyme; NEP, nuclear-encoded polymerase. Reproduced with modifications from [5] with permission.

Although the genetic diversity of some representative viroids has been characterized in previous work [9–11], CChMVd is the only viroid for which the rate of spontaneous mutation has been determined experimentally [4]. As such, it remains to be shown to what extent extremely high mutation rate is a more general property of viroids or, in contrast, is specific to CChMVd and closely-related viroids. CChMVd belongs to the family *Avsunviroidae*, the members of which replicate in plastids (mostly chloroplasts), where their single-stranded circular RNA is copied by a bacteriophage-like nuclear-encoded RNA polymerase (NEP) through a rolling-circle mechanism to yield linear oligomers [12, 13]. The latter are cleaved co-transcriptionally [14] by the embedded hammerhead ribozymes to yield monomers [15, 16], which are circularized by a tRNA ligase [17]. By convention, the (+) polarity is assigned to the most abundant strand, but the replication cycle is fully symmetric, i.e. identical for both polarities (Fig 1).

In contrast, members of the family *Pospiviroidae* are copied by RNA polymerase II in the nucleus, where rolling-circle replication of the circular (+) strand produces (–) oligomers, which are used directly for a second round of copying to yield (+) oligomers [18–20]. These replicative intermediates are then cleaved into (+) monomers by RNAse III [21] and circularized by DNA ligase I accepting RNA substrates [22] (Fig 1). Although analysis of genetic diversity suggested differences in replication fidelity between nuclear and chloroplastic viroids [9–11], recent work based on previously published deep sequencing data has posited that potato spindle tuber viroid (PSTVd), the type species of the family *Pospiviroidae*, might show extremely high copying error rates similar to those of CChMVd [23]. It therefore remains to be elucidated whether the two viroid families show different rates of spontaneous mutation. Importantly, these previous works have not disentangled the various factors contributing to genetic diversity, which include selection, mutational robustness, but also genetic drift and population structure among others, precluding an unbiased inference of mutation rates.

Here, we sought to quantify *in vivo* the mutation rate of one representative viroid of each family replicating in a common host. Typically, mutation rate estimation methods require highly controlled laboratory conditions as well as detailed information about the number of replication cycles elapsed [24], which makes them often unsuitable for measuring mutation rates *in vivo*. To circumvent this problem, it is possible to quantify the population frequency of lethal mutations [4]. This is because lethal mutations cannot be inherited, and hence, their frequency should equal the rate at which they are produced. As formalized by classical mutation-selection balance models, in haploid populations the equilibrium frequency of a deleterious mutation is *q* = *μ* / *s*, where *μ* is the rate of spontaneous mutation and *s* the selection coefficient [25]. Hence, whereas for slightly deleterious or neutral mutations (*s →* 0) the observed mutation frequency may strongly deviate from mutation rate (*q >> μ*), for highly deleterious mutations (*s →* 1) *q* will approach *μ*. Analysis of lethal or quasi-lethal mutations has been previously used for inferring the mutation rates of hepatitis C virus [26, 27], poliovirus [28], and human immunodeficiency virus 1 [29], in addition to CChMVd [4]. A complication of this approach, though, is that since lethal mutations have low population frequencies, sequencing must be carried out with both high depth and accuracy. Sanger sequencing is highly accurate but has limited depth, whereas standard next-generation sequencing (NGS) has extreme depth but low per-read accuracy. This problem has been solved recently by the development of methods that increase the accuracy of NGS by orders of magnitude, such as CircSeq and Duplex Sequencing (DS) [30, 31], which now permit a better characterization of viroid genetic diversity and mutation rates.

Here we have focused on DS because CirSeq demands high amounts of starting material and is thus impractical for viroid RNA obtained from infected tissue. DS reduces considerably sequencing mistakes by tagging and sequencing independently each of the two DNA strands multiple times, wherein true mutations are detected in the same position. Whereas DS does not allow removal of errors associated with reverse transcription and PCR, it can nevertheless strongly increase accuracy compared to conventional NGS by removing errors associated with sequencing. We have applied DS to the chloroplastic eggplant latent viroid (ELVd) and the nuclear PSTVd infecting eggplant to exclude possible biases caused by using different hosts. We found that, while ELVd showed an extremely high mutation rate similar to that of CChMVd [4], the mutation rate of PSTVd was lower and fell closer to the typical range of RNA viruses.

## Results

### DS provides in-depth sequence coverage while strongly reducing sequencing errors

**Fig 2:**
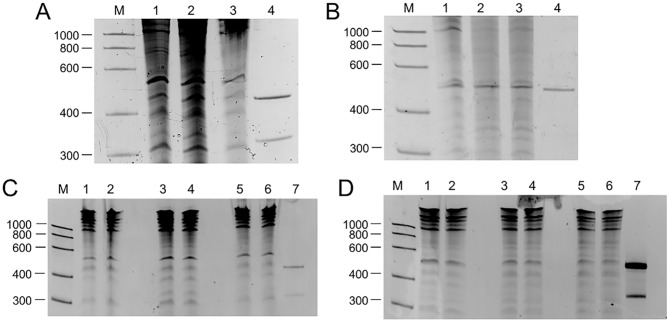
Analysis by denaturing PAGE of ELVd- and PSTVd-infected eggplant seedlings. **A**: ELVd at 12 mpi; **B**: PSTVd at 6 mpi. Lanes 1, 2 and 3: RNA preparations from the three plants inoculated with either ELVd or PSTVd. Lane 4: previously purified monomeric circular (upper bands) and linear (lower bands) RNAs of ELVd or PSTVd that served as a control of the viroid mobility (the monomeric linear PSTVd form is barely visible). **C** and **D**: ELVd and PSTVd extracts taken at 18 mpi. Lanes 1 and 2, 3 and 4, and 5 and 6: RNA preparation from ELVd or PSTVd-inoculated eggplant 1, 2 and 3, respectively. Lane 7, previously purified circular and lineal monomeric forms of PSTVd or ELVd (control of the viroid mobility). Lane M: RNA markers with their size (in nucleotides) indicated on the left. Gels were stained with ethidium bromide and are shown in inverted color to facilitate visualization.

For each ELVd and PSTVd, three eggplant seedlings were agro-inoculated with infectious plasmids containing head-to-tail dimeric inserts of the corresponding viroid cDNAs and total nucleic acids from upper non-inoculated leaves were extracted six months post-inoculation (mpi) for PSTVd and 12 mpi for ELVd. Subsequent fractionation with non-ionic cellulose [32] resulted in preparations enriched in RNAs with a high content in secondary structure, including viroid RNAs. The six individual preparations were separated by denaturing PAGE and the RNAs migrating between the markers of 400 and 600 nt containing the monomeric circular RNAs were eluted and recovered (Fig 2). To assess whether levels of genetic diversity varied among different viroid RNA forms, we also recovered the strands migrating between the markers of 600 and 1000 nt, which correspond to oligomeric viroid RNAs. The extracted RNAs were used for high-fidelity RT-PCR and sequenced by the DS method. The RT-PCR was performed with adjacent primers of opposite polarities to generate full-length products from the monomeric viroid circular (+) strands and the (–) oligomers, hence allowing us to sequence the entire viroid except for the primer regions. To control for errors associated with reverse transcription, PCR, and sequencing, we also performed DS of the PCR product obtained directly from a plasmid with a PSTVd insert, as well as of the RT-PCR products from (+) PSTVd and (+) ELVd RNAs transcribed *in vitro* using T7 or T3 RNA polymerases. The PCR products from plant extracts and the RT-PCR controls were tagged and analyzed in the same run, using a MiSeq Illumina sequencer. DS of the direct PSTVd PCR product with an average depth of 8071 reads per site yielded 16 total mutations in 2,808,781 bases read. Hence, assuming there was no variation in the plasmid template, the joint technical error rate of PCR and DS was 5.7 × 10^−6^. This rate increased to 3.9 × 10^−5^ and 4.4 × 10^−5^ in RT-PCR products from the *in vitro* transcripts of PSTVd and ELVd, respectively, showing the important contribution of RT to sequencing errors, although mutations arising during *in vitro* transcription were also probably present in these controls.

### ELVd and PSTVd display different mutation frequencies

**Fig 3:**
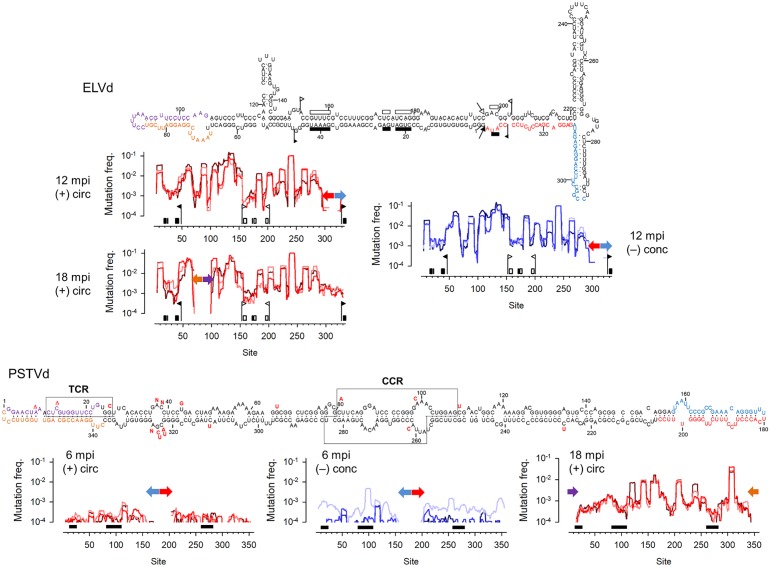
Genetic diversity of ELVd and PSTVd in eggplant as determined by DS. The viroid circular (+) strand sequences are depicted. Primers used for RT-PCR from samples taken at 6/12 mpi (run 1) are indicated with red and blue font in the sequences and those used for RT-PCR from samples taken at 18 mpi (run 2) are shown in orange and purple (see S1 Table for details). The skyline plots show mutation frequencies (total mutations/total reads per site) averaged over a 10-nucleotide sliding window to smooth the plot. Thick colored arrows in these graphs indicate the positions encompassed by RT-PCR primers, with the same color code as for the sequences. Red plots correspond to circular (+) strands and blue plots to (–) oligomers. Light, bright, and dark color correspond to plants 1, 2, and 3, respectively. In the ELVd sequence and skyline plots, key elements of the (+) hammerhead ribozyme are shown in black and those of the (–) hammerhead ribozyme in white. Flags indicate the regions encompassed by the hammerhead ribozymes of each polarity, arrows indicate self-cleavage sites in the sequences, and conserved motifs are indicated with boxes. By convention, ELVd sites are numbered starting from the (+) strand self-cleavage site. In the PSTVd sequence, the central conserved region (CCR) and terminal conserved region (TCR) are boxed and also indicated by black bars in the skyline plots. Red bases shown next to the PSTVd sequence indicate mutations previously shown to have lethal effects (see text for references). By convention, PSTVd sites are numbered starting from the left terminal loop. A site-by-site list of all mutations is available in S1 Dataset (6/12 mpi) and S2 Dataset (18 mpi).

Analysis of the three ELVd-infected plants yielded an average mutation frequency of (1.5 ± 0.3) × 10^−2^ for circular (+) strands and of (1.8 ± 0.3) × 10^−2^ for (–) oligomers (Table 1; S1 Dataset), confirming the extremely high genetic diversity of chloroplastic viroids. At an average sequencing depth of 764 reads/site/run, 264 of the 295 ELVd nucleotide sites examined (89%) were polymorphic. The genetic diversity was not uniformly distributed along the ELVd sequence, with peaks in regions encompassing sites 120–140 and 240–245, which map to a hairpin and a loop, respectively, in the secondary structure proposed *in vivo* for the monomeric ELVd (+) strand [33] (Fig 3). In contrast, relatively low diversity values were found in regions delimited by sites 69–79 and 95–103, which form the two strands of a base-paired stem, as well as an adjacent bulge. We also found lower-than-average diversity in the region encompassing sites 18–50, which maps to the (+) hammerhead ribozyme. Sites 152–180 and 188–200, which map to the (–) hammerhead ribozyme, also showed low diversity in circular (+) strands. Overall, there was an excellent correlation between per-site mutation frequencies in circular (+) strands and (–) oligomers of the same plant at the analyzed sampling time (Pearson correlation: *r* ≥ 0.952; *P* < 0.001). We also found that per-site mutation frequencies were significantly correlated among plants (*r* ≥ 0.785; *P* < 0.001), suggesting that genetic diversity was mainly driven by a deterministic mutation-selection balance.

**Table 1:** Summary of the DS data obtained for ELVd (12 mpi) and PSTVd (6 mpi) in eggplant^1^.

	ELVd						PSTVd					
	Plant 1		Plant 2		Plant 3		Plant 1		Plant 2		Plant 3	
	Circ (+)	Oligo (–)	Circ (+)	Oligo (–)	Circ (+)	Oligo (–)	Circ (+)	Oligo (–)	Circ (+)	Oligo (–)	Circ (+)	Oligo (–)
**Total bases read**	172,111	136,598	211,559	248,762	134,976	447,920	1,657,249	1,294,481	1,395,515	1,531,703	1,065,790	1,236,993
**Mean depth**	583	463	717	843	458	1518	5434	4244	4575	5022	3494	4056
**Unique mutations**^2^	178	173	200	212	161	279	178	284	124	107	101	62
**Total mutation count**^3^	1902	2100	2614	3976	2985	10,616	298	914	198	185	141	103
**Mutation frequency**^4^ **(× 10^3^)**	11.1	15.4	12.4	16.0	22.1	23.7	0.180	0.706	0.142	0.121	0.132	0.083

^1^A list of all mutations is provided in S1 Dataset

^2^Number of different mutations found (presence/absence)

^3^Number of mutation reads

^4^Total mutation count divided by total bases read

In PSTVd, we found variation in 262 of the 304 sites examined (86%) at an average sequencing depth of 4471 reads/site/run. Hence, a greater depth was required to sample a number of polymorphic sites similar to that found for ELVd. The average frequency of mutations was two orders of magnitude lower in PSTVd than in ELVd, with values of (1.5 ± 0.2) × 10^−4^ and (3.0 ± 2.0) × 10^−4^ for circular (+) strands and (–) oligomers, respectively (Table 1). The higher diversity of (–) oligomers was driven by an anomalously high mutation frequency in plant 1 (7.1 × 10^−4^). Importantly, mutation frequency was significantly higher in sequences obtained from PSTVd-infected plants than in control sequences derived from the *in vitro* transcript, indicating that most sequence variants detected *in vivo* were real and not RT-PCR-sequencing artifacts (Fisher test: *P* < 0.001 in all six runs). As for ELVd, we found significant correlations between the per-site mutation frequencies of circular (+) strands and (–) oligomers in plant 2 (*r* = 0.551; *P* < 0.001) and plant 3 (*r* = 0.735; *P* < 0.001). This correlation, albeit still significant, was much lower in plant 1 (*r* = 0.228; *P* < 0.001), with the (–) oligomers of this plant showing a distribution of mutations across sites discordant with the other five runs (Fig 3). Excluding this anomalous set, we also observed correlations between per-site mutation frequencies from different plants, albeit lower than those found for ELVd (*r* ≥ 0.468; *P* < 0.001). This result could be explained by the greater difficulty of reproducibly sampling rarer genetic variants, or could indicate that random genetic drift has a stronger influence on genetic diversity in PSTVd than for ELVd. Finally, no major diversity peaks were found in PSTVd except for position 117 at the terminus of an A-rich sequence in the pathogenicity domain of the secondary structure proposed *in vivo* for the monomeric PSTVd (+) strand [34, 35] (Fig 3). This region had a marked tendency to point insertions/deletions, possibly resulting from polymerase slippage.

The most abundant types of mutations in ELVd sequences from circular (+) strands were transitions (75.4 ± 4.6% of all mutations), followed by transversions (21.7 ± 4.5%) and point insertions (2.5 ± 0.1%), whereas point deletions were the rarest type (0.4 ± 0.1%). C-to-U, G-to-A, and U-to-C substitutions were found at similar frequencies, whereas A-to-G changes were slightly less frequent. A very similar pattern was found for sequences derived from (–) oligomers (Table 2). In contrast, the mutational spectrum was markedly different in PSTVd, with 50.5 ± 2.0% transitions, 40.8 ± 1.6% transversions, 3.6 ± 1.1% insertions, and 5.2 ± 0.6% deletions in circular (+) strands, versus 56.5 ± 3.5%, 31.5 ± 6.2%, 6.4 ± 2.7%, and 5.6 ± 0.6% in (–) oligomers, respectively. Contrarily to ELVd, we found clear differences among transition types in PSTVd, such that C-to-U > G-to-A > U-to-C > A-to-G in circular (+) strands, whereas (–) oligomers showed a different pattern (G-to-A > A-to-G > U-to-C ≈ C-to-U; Table 2). The different mutational spectrum of PSTVd (+) and (–) strands was explained in part by reverse complementarity, i.e. C-to-U > G-to-A in (+) strands as opposed to G-to-A > C-to-U in (–) strands, and U-to-C > A-to-G in (+) strands as opposed to A-to-G > U-to-C in (–) strands.

**Table 2:** Mutational spectrum (% total mutations) of ELVd (12 mpi) and PSTVd (6 mpi).

	ELVd		PSTVd	
	Circ (+)	Oligo (–)	Circ (+)	Oligo (–)
**C-to-U**	24.3 ± 2.4	24.6 ± 1.2	25.7 ± 4.2	2.8 ± 2.1
**G-to-A**	23.8 ± 1.5	20.7 ± 1.5	13.3 ± 3.6	31.8 ± 3.2
**U-to-C**	18.5 ± 0.8	17.0 ± 0.6	7.9 ± 2.0	3.0 ± 1.0
**A-to-G**	8.7 ± 1.2	10.6 ± 0.8	3.5 ± 0.8	18.9 ± 4.7
**G-to-U**	3.5 ± 0.8	4.4 ± 0.9	21.7 ± 2.7	0.43 ± 0.43
**C-to-A**	0.89 ± 0.22	1.2 ± 0.2	4.9 ± 2.5	22.8 ± 3.9
**U-to-G**	3.9 ± 0.3	6.7 ± 0.2	0.37 ± 0.37	1.1 ± 0.6
**A-to-C**	0.32 ± 0.27	0.43 ± 0.31	1.9 ± 1.0	0.10 ± 0.10
**U-to-A**	6.9 ± 1.7	6.3 ± 1.1	1.7 ± 0.3	2.9 ± 1.6
**A-to-U**	5.7 ± 3.0	5.1 ± 1.9	3.3 ± 0.1	0.25 ± 0.25
**G-to-C**	0.40 ± 0.16	0.13 ± 0.04	3.7 ± 1.2	0.65 ± 0.65
**C-to-G**	0.03 ± 0.03	0.13 ± 0.03	3.3 ± 1.5	3.3 ± 1.3
**Insertion**	2.5 ± 0.1	2.5 ± 0.2	3.6 ± 1.2	6.4 ± 2.7
**Deletion**	0.44 ± 0.12	0.13 ± 0.04	5.2 ± 0.6	5.6 ± 1.2

### The mutation rate of PSTVd is lower than that of ELVd

**Fig 4:**
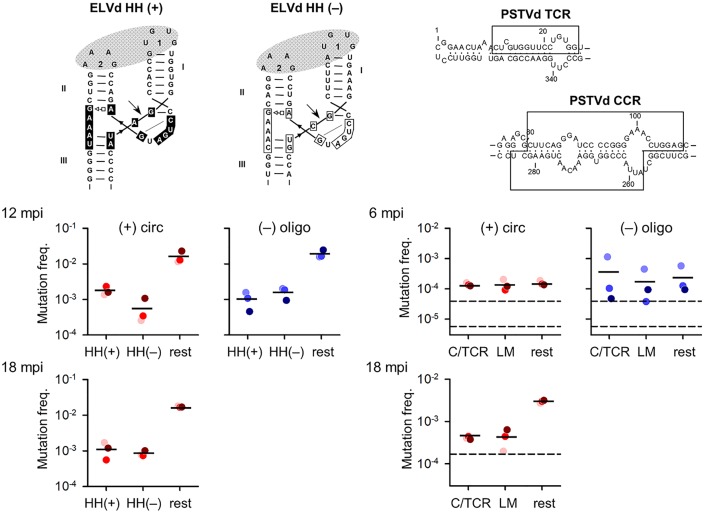
Mutations in essential motifs of the ELVd hammerhead ribozymes and in PSTVd conserved regions. A schematic representation of the ELVd hammerhead ribozymes (HH) is shown on top for both (+) and (–) strands, with the nucleotides forming the catalytic core within boxes, arrows indicating self-cleavage sites, and grey ovals denoting interactions between loops. Mutation frequencies for the catalytic core of each hammerhead ribozyme (15 sites) and the rest of the ELVd sequence are shown below, indicating sampling time (run 1: 6/12 mpi; run 2: 18 mpi) and RNA forms [red: circular (+) strands; blue: (–) oligomers]. For PSTVd, the CCR and TCR are depicted on top. Below are shown the mutation frequencies for the CCR/TCR sites (C/TCR), the set of 23 lethal mutations (LM) mostly mapping outside CCR/TCR, and the rest of the PSTVd sequence. For each viroid, plants 1, 2 and 3 are shown in light, bright and dark color dots, respectively. Horizontal bars indicate mean mutation frequencies. In the PSTVd graphs, the dashed horizontal lines indicate the error rates associated with *in vitro* transcription, RT, and PCR. The error associated with PCR alone is also shown for the 6/12 mpi run (lower dashed line).

To estimate the ELVd mutation rate by the lethal mutation method we focused on the hammerhead ribozymes, which mediate self-cleavage of the linear oligomers and are hence essential for completing the replication cycle. The hammerhead ribozyme consists of a central catalytic core of 15 nucleotides flanked by three double helices [16, 36] (Fig 4). The core nucleotides are required for the catalytic activity of the ribozyme [36–38] and, since the vast majority of mutations at these positions inactivate self-cleavage activity, the 15 sites can be used for mutation rate inference using the lethal mutation method, as shown previously [4]. In circular (+) strands average mutation frequencies were (1.8 ± 0.3) × 10^−3^ for the (+) hammerhead and (0.6 ± 0.3) × 10^−3^ for the (–) hammerhead, whereas in (–) oligomers the frequencies were (1.1 ± 0.3) × 10^−3^ for the (+) hammerhead and (1.6 ± 0.3) × 10^−3^ for the (–) hammerhead ribozymes. Such reduction of one order of magnitude in diversity compared with the rest of the sequence was expected, because mutations falling at these essential domains should tend to be lethal and hence leave little or no progeny. Therefore, mutation frequencies in these domains should resemble the rate of spontaneous mutation, as opposed to those in the rest of the sequence. Averaging the above values, the estimated rate of spontaneous mutation of ELVd was (1.3 ± 0.3) × 10^−3^, or roughly one mutation every 800 bases.

We adopted the same mutation rate estimation approach for PSTVd. To do this, we focused on specific sites forming the central conserved region (CCR) and the terminal conserved region (TCR). These regions mediate key functions including replication for the CCR, or are presumed to play alternative essential roles (yet unknown) for the TCR [39]. Hence, most mutations at these sites should have highly deleterious or lethal effects. We also focused on a set of 23 different single-base substitutions previously reported to impair PSTVd infectivity [40–44]. In circular (+) strands, average mutation frequencies were (1.4 ± 0.1) × 10^−4^ for the CCR/TCR regions and (1.4 ± 0.3) × 10^−4^ for the set of previously described lethal mutations (Fig 4). We found similar values in (–) oligomers, except that variance was higher and that plant 1 showed a higher value, as discussed above. Therefore, the estimated rate of spontaneous mutation of PSTVd was 1.4 × 10^−4^ or roughly one mutation every 7000 bases. Unexpectedly, mutation frequencies at these essential sites were not lower than those obtained for the rest of the PSTVd sequence. It is possible that most mutations in PSTVd are highly deleterious or lethal, regardless of whether they map or not to these specific regions, meaning that PSTVd would show very low mutational tolerance. It is also possible that genetic diversity accumulated at low rates due to slow replication, such that few polymorphisms were produced at 6 mpi. Alternatively, the actual PSTVd mutation rate might be lower than 1.4 × 10^−4^ and the noise introduced by sequencing errors could have precluded us from measuring this lower value. However, our overall sequencing error rate as determined using a PSTVd RNA transcribed *in vitro* was 3.6-fold lower (3.9 × 10^−5^) than the estimated PSTVd mutation rate (the error rates estimated specifically for the CCR/TCR and the set of 23 predefined mutations being 2.0 × 10^−5^ and 6.6 × 10^−5^, respectively). Notice that this probably represents an upper-limit to the actual sequencing error rate, because *in vitro* transcription is an error-prone process that may have contributed mutations in our control assays, but not in actual sequences from plants.

### Analysis of plants after 18 months of viroid replication

We performed a second set of experiments from the same plants at 18 mpi to address whether viroid diversity depended on sampling time and/or sequencing run. The RNA extraction procedure was identical except that we focused only on monomeric circular RNAs (i.e. migrating between the markers of 400 and 600 nt; Fig 2C and 2D). As above, the RT-PCR was performed with adjacent primers of opposite polarities producing full-length products from monomeric circular (+) RNAs, but annealing at different regions in order to cover the portions of viroid sequence that were not analyzed in the first run (see Fig 3). This new run included six PCR products (three plants, two viroids) as well as controls of RT-PCR products from (+) PSTVd and (+) ELVd RNAs transcribed *in vitro*. The three ELVd-infected plants yielded an average mutation frequency of (1.45 ± 0.04) × 10^−2^, which is nearly identical to the value obtained in the first run at 12 mpi (Table 3). Furthermore, the distribution of mutations along the ELVd sequence was also highly similar between the two time points (Fig 3; within-plant Pearson correlation between per-site mutation frequencies at 12 and 18 mpi: *r* ≥ 0.795), confirming that ELVd reached a deterministic mutation-selection balance.

**Table 3:** Summary of the DS data obtained for ELVd and PSTVd (+) monomeric circular RNAs from eggplant^1^.

	ELVd			PSTVd		
	Plant 1	Plant 2	Plant 3	Plant 1	Plant 2	Plant 3
**Total bases read**	609,332	1,149,128	954,810	1,478,180	867,547	560,781
**Mean depth**	2073	3909	3248	4723	2772	1792
**Unique mutations**^2^	351	284	322	345	296	209
**Total mutation count**^3^	9330	16303	13252	3880	2432	1658
**Mutation frequency**^4^ **(× 10^3^)**	15.3	14.2	13.9	2.62	2.80	2.96

^1^A list of all mutations is provided in S2 Dataset

^2^Number of different mutations found (presence/absence)

^3^Number of mutation reads

^4^Total mutation count divided by total bases read

At 18 mpi, the average frequency of mutations in PSTVd was (2.8 ± 0.1) × 10^−3^, a value an order of magnitude higher than at 6 mpi but still five times lower than for ELVd (Table 3). In addition to position 117 at the terminus of an A-rich sequence (which already showed a high frequency of point insertions/deletions at 6 mpi) at 18 mpi, we found other single-nucleotide polymorphisms (G143A, U161C, C167A, U209del, and U309A) that independently arose at high population frequencies (> 5%) in the three plants (Fig 3). Removal of these few sites from the analysis reduced the average mutation frequency by threefold, i.e. (9.0 ± 1.1) × 10^−4^. We also found that the distribution of mutations along the sequence was markedly different at 6 and 18 mpi (within-plant correlation between per-site mutation frequencies: *r* ≤ 0.131; Fig 3). Contrarily, the per-site mutation frequencies from the three plants were highly correlated at 18 mpi (*r* ≥ 0.917; Fig 3). Hence, in contrast to the earlier analysis in which random processes such as genetic drift appeared to play an important role in PSTVd genetic diversity, sequences obtained at 18 mpi were more consistent with a deterministic mutation-selection balance, similar to the pattern found for ELVd. Therefore, PSTVd accumulated relatively low levels of diversity at 6 mpi and showed an unexpectedly slow onset of mutation-selection balance.

As above, we estimated the spontaneous mutation rate of ELVd by focusing on the 15 central catalytic core nucleotides of each hammerhead ribozyme. Average mutation frequencies were (1.2 ± 0.3) × 10^−3^ for the (+) hammerhead and (0.9 ± 0.1) × 10^−3^ for the (–) hammerhead, or approximately one mutation every 1000 bases copied. These values are similar to those obtained at 6 mpi (Fig 4). For PSTVd, we again focused on CCR/TCR, as well as on the set of 23 single-point mutations previously reported to impair PSTVd infectivity. Average mutation frequencies were (4.1 ± 0.2) × 10^−4^ for the CCR/TCR and (4.5 ± 1.3) × 10^−4^ for the set of previously described lethal mutations (Fig 4). As opposed to the results obtained at 6 mpi, these values were six-fold lower on average than those obtained for the rest of the PSTVd sequence (Fig 3). This supports the conclusion that the accumulation of diversity was restricted specifically at these sites by the strongly deleterious/lethal effects of mutations. On the other hand, mutation frequencies at the CCR/TCR and for the set of 23 predefined mutations were threefold higher at 18 than at 6 mpi, which was unexpected assuming that these mutations were lethal. This discrepancy could be in part explained by a higher sequencing error rate in this run. The in-vitro transcribed PSTVd control showed a mutation frequency of 1.7 × 10^−4^ (172 mutations in 1,014,425 bases read) in the CCR/TCR and of 1.6 × 10^−4^ (44 mutations in 271,663 bases read) for the set of 23 predefined mutations, versus 2.0 × 10^−5^ and 6.6 × 10^−5^ in the previous experiment, respectively. By subtracting the corresponding error rates obtained in the 18 mpi run, the estimated net mutation frequencies were 2.4 × 10^−4^ and 2.9 × 10^−4^ for the CCR/TCR and the predefined set, respectively, suggesting approximately one mutation every 3800 bases.

## Discussion

Owing to their lethality or quasi-lethality, mutations in the catalytic core of ELVd hammerhead ribozymes as well as in the CCR/TCR and some specific sites of PSTVd should have a very small number of rounds of copying to accumulate. Specifically, mutations falling at the central catalytic core of the hammerhead ribozyme should be able to survive for 0 to 2 rounds of copying, depending on the polarities of the sequenced strand and of the mutated hammerhead ribozyme (Fig 1). This makes them an excellent target for mutation rate inference by the lethal mutation approach, and a similar argument should hold for the CCR/TCR and the set of PSTVd mutations previously shown to inactivate infectivity. For instance, changes inactivating the ELVd (+) hammerhead ribozyme should prevent production of circular (+) RNA, implying that these mutations should not be found in the catalytic core of the (+) hammerhead ribozyme in sequences derived from the circular (+) strand template. In contrast, we found mutations at a frequency in the order of 10^−3^ in these sequences, a value not attributable to RT-PCR-sequencing errors because the latter were two orders of magnitude less frequent. Yet, at least two other explanations are possible. First, some mutations may have resulted from RNA editing or spontaneous RNA damage (*in vivo*, or during the extraction process). RNA damage appears more likely in the single-stranded circular (+) RNA than in the in (–) oligomers, a fraction of which could be forming double-stranded complexes. Second, the hammerhead ribozyme located in the 5´-end repeat of the (+) oligomer should not be required for cleavage and hence may incorporate loss-of-function mutations, as opposed to the other oligomer repeats.

We have found a mutation rate for ELVd (1/100 to 1/800) relatively similar to that of CChMVd (1/400) and, hence, our results suggest that an extremely fast mutation is shared by at least two of the four chloroplastic viroids. In contrast, the mutation rate of PSTVd was 4–8 times lower and more similar to those of RNA viruses [3]. This marked difference is probably at the origin of the higher genetic diversity of chloroplastic viroids compared with their nuclear counterparts. RNA polymerase II has proofreading capacity [45] and its estimated misincorporation rate in *Caenorhabditis elegans* is 4 × 10^−6^, the most frequent errors being C-to-U, followed by G-to-U [46]. Interestingly, these were also the two most frequent mutation types in PSTVd (+) circular strands, although the overall mutation rate of PSTVd was much higher than the estimated RNA polymerase II misincorporation rate. This suggests that the fidelity of RNA polymerase II is strongly reduced when the enzyme is forced to accept viroid RNA as template instead of nuclear DNA. In contrast to PSTVd, chloroplastic viroids are thought to be copied by a bacteriophage-like NEP with a lower fidelity than RNA polymerase II [47, 48]. In addition to differences in replication fidelity, we cannot discard other factors that could contribute to explaining differences in mutation rates, such as RNA editing [49] and spontaneous RNA damage. The more open secondary structure of chloroplastic viroids could increase susceptibility to RNA damaging agents. Furthermore, mutagenic free radicals resulting from electron transduction during photosynthesis, as well as unbalanced nucleotide pools, may also contribute to increased mutation rates in the chloroplast. Finally, in addition to differences in replication fidelity and/or RNA damage, chloroplastic and nuclear viroids may also exhibit different tolerance to mutations [50]. We found that, at 6 mpi, PSTVd mutation frequencies showed low heterogeneity along the sequence, with few peaks of diversity and no apparent differences between the CCR/TCR and other viroid regions. However, higher diversity was apparent in some PSTVd regions at 18 mpi.

According to the RNA world hypothesis, RNA preceded DNA as the carrier of genetic information during early stages of life. Indirect evidence supporting the existence of an RNA world is provided by ribozymes, which include the hammerhead structures found in chloroplastic viroids and in viroid-like satellite RNAs. It has been suggested that their small size, circularity, high G+C content, lack of protein-coding ability, and, specially, the catalytic activity associated to ribozymes, make these minimal replicons candidates for being relics of early life-forms [5, 8]. An important consequence of error-prone copying in early replicons is the existence of a limit to genome complexity, as genomes over a certain size would incur in an excessive mutational load. This limit would prevent the evolution of new functions, including repair mechanisms, thereby trapping RNA genomes in an evolutionary dead-end, a problem known as Eigen’s paradox [51]. This constraint predicts a negative correlation between mutation rate and genome size, although such correlation may have other explanations, including random genetic drift [52] and mutation rate optimization [53]. Whereas there is strong evidence for such a negative correlation among viruses and bacteria [2, 3, 53, 54], viroids, are not self-replicating entities and hence should be subject to different contraints. Except for the possible role of secondary structure, factors determining the mutation rate of nuclear viroids are mainly controlled by the host, implying that lower mutation rates may not be evolutionarily accessible to them. Whereas extremely high mutation rates may situate chloroplastic viroids close to Eigen´s error threshold and may hence impose limits to the evolution of larger sequences, this does not seem to be the case for PSTVd and, probably, other nuclear viroids.

## Materials and methods

### Plant inoculation and RNA fractionation

Eggplant seedlings (*Solanun melongena* cv. ‘Redonda morada’) were PSTVd- or ELVd-agro-inoculated 6 and 12 months, respectively, before RNA extraction (run 1), and 18 months before RNA extraction (run 2). Total nucleic acids were extracted by grinding systemic leaves in buffer-saturated phenol, and then fractionated on non-ionic cellulose (CF11; Whatman) with STE (50 mM Tris-HCl, pH 7.2, 100 mM NaCl, 1 mM EDTA) containing 16% ethanol [32]. The resulting preparations, enriched in RNAs with a high content in secondary structure including viroid RNAs, were electrophoresed in denaturing 5% polyacrylamide gels containing 89 mM TBE (Tris-Borate-EDTA) and 8 M urea. The gels were stained with ethidium bromide and the viroid circular RNA (migrating between the linear RNA markers of 400 and 600 nt) and the viroid oligomeric forms (migrating between the linear RNA markers of 600 and 1000 nt), were excised, eluted overnight with 10 mM Tris-HCl, pH 7.5 containing 1 mM EDTA and 0.1% SDS, and recovered by ethanol precipitation.

### Preparation of controls

The substrate for the ELVd control was the dimeric product resulting from *in vitro* transcription driven by the T7 promotor of a recombinant plasmids containing a dimeric head-to-tail ELVd-cDNA insert of the reference variant ELVd-2 (GenBank AJ536613). For the PSTVd control, the substrate was the monomeric product resulting from *in vitro* transcription driven by the T3 promotor of a recombinant plasmid containing a monomeric PSTVd-cDNA insert of variant RG1 (GenBank U23058) opened between positions C1-G2 flanked by a modified version of the hammerhead ribozyme of tobacco ringspot virus satellite RNA and a modified version of the ribozyme of hepatitis delta virus minus RNA strand [20, 22]. The resulting unit-length transcripts, purified by denaturing PAGE and subsequent elution, were added to leaves of healthy eggplant homogenized in buffer-saturated phenol and the RNA extraction was continued as indicated in the previous section in order to prepare the controls under conditions mimicking those of infected samples.

### Reverse transcription and polymerase chain reaction

Prior to reverse transcription, all samples and controls were treated with the TURBO DNA-free kit (Ambion) to remove any DNA contamination following manufacturer´s instructions. ELVd circular (+) and oligomeric (–) RNAs purified from infected tissue were reverse transcribed for 1 h at 42°C with AccuScript Hi-Fi reverse transcriptase (Agilent) and primer RF-1298 (run 1) or RF-1405 (run 2) for the circular forms, and with primer RF-1299 for the oligomers (see S1 Table for details). The cDNA products were PCR-amplified with Phusion High-Fidelity DNA Polymerase (Thermo Scientific) and adjacent primers RF-1298 and RF-1299 (run 1) or RF-1404 and RF-1405 (run 2), using the following program: 1 min at 98°C, 35 cycles of 15 s at 98°C, 20 s at 66°C or 62°C (run 1 or 2, respectively), and 30 s at 72°C, with a final extension of 2 min at 72°C. The ELVd control RNA was reverse transcribed with primer RF-1298 (run 1) or RF-1405 (run 2) and PCR-amplified with this primer and primer RF-1299 (run 1) or RF-1404 (run 2). PSTVd RNA purified from infected tissue was reverse transcribed with primer RF-1242 (run 1) or RF-1406 (run 2) for circular forms and RF-1359 for oligomers. The cDNA products were PCR-amplified with adjacent primers RF-1242 and RF-1359 (run 1), or RF-1406 and RF-1407 (run 2) using the following program: 1 min at 98°C, 35 cycles of 15 s at 98°C and 20 s at 72°C, and a final extension of 2 min at 72°C. For run 1, the PSTVd RNA control was reverse transcribed with primer PSTVd-rev and PCR-amplified with this primer and primer PSTVd-fw of the PSTVd RG1 variant. For run 2, this control was reverse transcribed with primer RF-1406 and PCR-amplified with this primer and primer RF-1407. For PSTVd extracts taken at 18 mpi, we observed a minor additional PCR band and we excised the band of interest by running a 5% non-denaturing polyacrylamide gel.

### Duplex sequencing

This technique increases per-read accuracy by orders of magnitude compared to standard Illumina sequencing, using adapters that have random yet complementary double-stranded nucleotide sequences [55]. Since the probability of two molecules being labeled with the same adapter sequence is vanishingly small, these molecular tags can be used to identify reads originating from each individual strand of DNA in the sequencing output and calculation of a consensus sequence for each of these individual strands, hence allowing removal of sequencing errors. DS adapters were constructed by annealing two oligonucleotides, one of which contained a 12-nt single-stranded randomized sequence tag. Annealed oligonucleotides were extended using the Klenow fragment, digested with a specific restriction endonuclease to produce cohesive ends, and annealed to viroid RT-PCR products for library preparation, following previously described protocols [31]. Given the small size of viroids, no template fragmentation was required. A library was prepared to identify each RT-PCR product and run on an Illumina MiSeq machine sequencer. Sequencing of direct PCR controls was made on a separate run. FastQ files were processed with the DS software pipeline (https://github.com/loeblab/Duplex-Sequencing) using BWA 0.6.2, Samtools 0.1.19, Picard-tools 1.130 and GATK 3.3–0, and GenBank accessions AJ536613 and AJ634596 as reference sequences for ELVd and PSTVd, respectively. After parsing of tags, the first 200 bases of each read were selected to increase accuracy, and initial alignment and single stranded consensus sequence (SSCS) were assembled, followed by duplex consensus sequence (DCS) assembly. The DCS outputs were finally realigned to the reference sequence to count mutations. Previously defined default parameters were used for this process [31].

### Mutation rate estimation by the lethal method

As described previously, the frequency of lethal mutations in a population should equal the rate at which these mutations are produced [4, 26–29]. For ELVd, we assumed that all mutations in the hammerhead ribozyme core sites defined in Fig 4 should be lethal [4], and the same assumption was made for the PSTVd CCR/TCR sites analyzed. The overall mutation rate was simply estimated as $\mu =\frac{\sum_{i=1}^{T}N_{i}}{\sum_{i=1}^{T}C_{i}}$, where *N*_*i*_ is the number of mutations at site *i*, *C*_*i*_ is sequencing coverage at site *i*, and *T* is the number of sites analyzed. For the 23 mutations that were previously reported to have lethal effects in PSTVd [40–44], the estimation was more complicated because for most of the sites only one or two of the three possible base substitutions could be used for mutation rate estimation, as the other substitutions were not reported lethals. To account for this, the mutation rate was estimated as $\mu =\frac{\sum_{i=1}^{T}N_{i}}{\sum_{i=1}^{T}\sum_{j=1}^{k}\frac{\rho_{j}}{\rho_{i}}C_{i}}$, where *ρ*_*j*_ is the contribution of the specific mutation considered to the total mutation spectrum of PSTVd, *k* is the number of different lethal substitutions at site *i* (*k* = 1, 2, or 3) and *ρ*_*i*_ is the contribution of the three possible base substitutions at this site to the total mutational spectrum. These coefficients are provided in Table 2 for sequences obtained from circular (+) and oligomeric (–) forms. For instance, if at a given site only C-to-U substitutions were lethal, *k* = 1 and, for sequences from circular (+) forms, we used *ρ*_*j = 1*_ = 25.7 (i.e. the percentage of C-to-U mutations in the total spectrum) and *ρ*_*i*_ = 25.7 + 4.9 + 3.3 (i.e. the percentages of C-to-U, C-to-A, and C-to-G mutations). Notice that this formula could also be used for estimating mutation rates in the PSTVd CCR/TCR and ELVd hammerhead ribozymess more precisely, but this was not necessary as long as base composition and mutational spectra are similar for these regions and the rest of the viroid sequence.

## Supporting information

S1 Table Primers used for RT-PCR. (DOCX)

S1 Dataset List of all mutations sequenced for ELVd and PSTVd for run 1, indicating the genome position, type of mutation, relevant regions (HHs and CCR/TCR) and previously identified lethal mutations for PSTVd. Sites excluded from the analysis are indicated with a hash character, and the primer region is indicated.
(XLSX)

S2 Dataset List of all mutations sequenced for ELVd and PSTVd for run 2. (XLSX)
