# Supplementary material for: Different rates of spontaneous mutation of chloroplastic and nuclear viroids as determined by high-fidelity ultra-deep sequencing
Source: PLoS Pathog. 2017 Sep 14;13(9):e1006547. doi: 10.1371/journal.ppat.1006547 (PMC5614642; doi:10.1371/journal.ppat.1006547)
Supplement: S1 Table — (DOCX) [file ppat.1006547.s001.docx]

| **Primers and polarity^a^** | **Sequence** | **Positions^b^** | **Run** | **Use** |
| --- | --- | --- | --- | --- |
| RF–1298 (r) | TATCTCTCCTGGAAGRCCGG^c^ | 313–294 | 1 | RT–PCR ELVd circular (+) RNA, control |
| RF–1299 (f) | GAGGACGACCTCTCCCCATA | 314–333 | 1 | RT–PCR ELVd oligomer (-) RNA |
| RF-1405 (r) | CCTTHAAACGTTCCTCCAAG^d^ | 86-105 | 2 | RT-PCR ELVd circular (+), control |
| RF-1404 (f) | ACGAATCCTCCGAATTTA | 68-85 | 2 | PCR ELVd circular (+), control |
| RF–1242 (r) | AAACCCTGTTTCGGCGGGAATTAC | 179–156 | 1 | RT–PCR PSTVd circular (+) RNA |
| RF–1359 (f) | TCACCCTTCCTTTCTTCGGGTGTCC | 180–204 | 1 | RT–PCR PSTVd oligomer (-) RNA |
| PSTVd-rev (r) | AGGAACCAACTGCGGTTCCAAG | 359–338 | 1 | RT–PCR PSTVd control |
| PSTVd-fw (f) | CGGAACTAAACTCGTGGTTCCTG | 1–23 | 1 | PCR PSTVd control |
| RF-1406 (r) | GAGGAACCAACTGCGGTTCCAAG | 338-1 | 2 | RT-PCR PSTVd circular (+), control |
| RF-1407 (f) | GGAACTAAACTCGTGGTTCCTG | 2-23 | 2 | PCR PSTVd circular (+), control |

**Table S1.** Primers used for RT-PCR.

^a^f, forward; r, reverse

^b^Numbering refers to plus polarity

^c^R refers to G and A

^d^H refers to A, C and T
